# PMcardio‐Assisted ECG Interpretation in Non‐ST‐Elevation Acute Coronary Syndrome: A Case Report

**DOI:** 10.1002/ccr3.73517

**Published:** 2026-09-11

**Authors:** Kim Wouters, Atif Qureshi, Muhammad Sharjeel Abbas, Ramzi Zeidan, Priyavardhan Mishra, Murtaja Satea, Rinaldo Lauwers, Bipin Chaurasia

**Affiliations:** ^1^ Department of Psychiatry, Psychology and Neuroscience King's College London London UK; ^2^ Division of Cardiothoracic Surgery University of Iowa Carver College of Medicine Iowa City Iowa USA; ^3^ Gomal Medical College Khyber Medical University Peshawar Pakistan; ^4^ Medical School University of Debrecen Debrecen Hungary; ^5^ Dr. D. Y. Patil Medical College, Hospital and Research Centre Navi Mumbai India; ^6^ Department of Neurosurgery, College of Medicine University of Warith Al‐Anbiyaa Karbala Iraq; ^7^ Department of Emergency Medicine Onze Lieve Vrouw Ziekenhuis (OLV Hospital) Aalst Belgium; ^8^ Department of Neurosurgery Neurosurgery Clinic Birgunj Nepal

**Keywords:** artificial intelligence, chest pain, myocardial infarction, PMcardio

## Abstract

A 53‐year‐old man presented with exertional chest pain. The electrocardiogram and PMcardio smartphone application flagged ischemic changes. Urgent coronary angiography revealed a thrombotic occlusion of the proximal right coronary artery, treated by percutaneous coronary intervention with two drug‐eluting stents and restoration of coronary flow.

## Key Clinical Message

This case highlights the potential role of AI‐assisted ECG analysis in recognition of myocardial ischemia, particularly in inconclusive initial findings. AI tools may enhance diagnostic accuracy and support clinical decision‐making in emergency settings. However, they should be used as complementary tools alongside clinical assessment, ECG interpretation, and cardiac biomarkers.

## 1 Introduction

Acute coronary syndrome (ACS) continues to be one of the leading causes of disease and deaths all over the world and one of the most common reasons for presentation to the emergency department [1]. Despite the fact that chest pain is the most common symptom among patients, diagnosing ACS can often prove to be difficult.

Despite advancements in the approach used in the diagnosis of this condition, about 5%–10% of the population that presents chest pain are finally diagnosed with ACS [1, 2]. Early detection is crucial as late diagnosis results in poor prognosis. This, however, becomes difficult in cases where the changes observed in the electrocardiogram are slight and the initial biomarkers are either negative or slightly positive [1, 2]. This situation is common in non‐ST‐segment elevation, where classic criteria for ACS may be absent even though there is coronary artery disease.

In recent times, the emergence of artificial intelligence (AI) based diagnostic tools is increasingly being seen as an effective support to the conventional methods of diagnosis. The PMcardio smartphone application analyzes a photographed 12‐lead ECG and has shown satisfactory diagnostic accuracy for major ECG abnormalities in primary care [3], while a dedicated occlusion myocardial infarction (OMI) model within the application has been evaluated for the detection of acute coronary occlusion [4]. Such tools could prove helpful in the diagnosis of myocardial ischemia.

## 2 Case History/Examination

### 2.1 History of Presentation

A 53‐year‐old obese man presented himself at the emergency department with a 2‐week history of progressive exertional chest pain. The character of the pain was described as a squeezing chest pain radiating to his left arm and jaw. The pain was aggravated by physical exertion and was not relieved by rest or analgesics.

### 2.2 Past Medical History

The patient was previously diagnosed with hypertension, hyperlipidemia, and NSTEMI. He also had a history of familial cardiac illness. The medications that he was using include paracetamol, L‐thyroxine, and pantoprazole.

### 2.3 Vitals and Examination

The patient was hypertensive upon admission with a blood pressure of 176/105 mmHg. The heart rate was 66 beats per minute, temperature was 36.6°C, and oxygen saturation was 98% in room air. There were no signs of cardiopulmonary pathology or heart failure.

## 3 Methods (Differential Diagnosis, Investigations and Treatment)

### 3.1 Investigations

**FIGURE 1:**
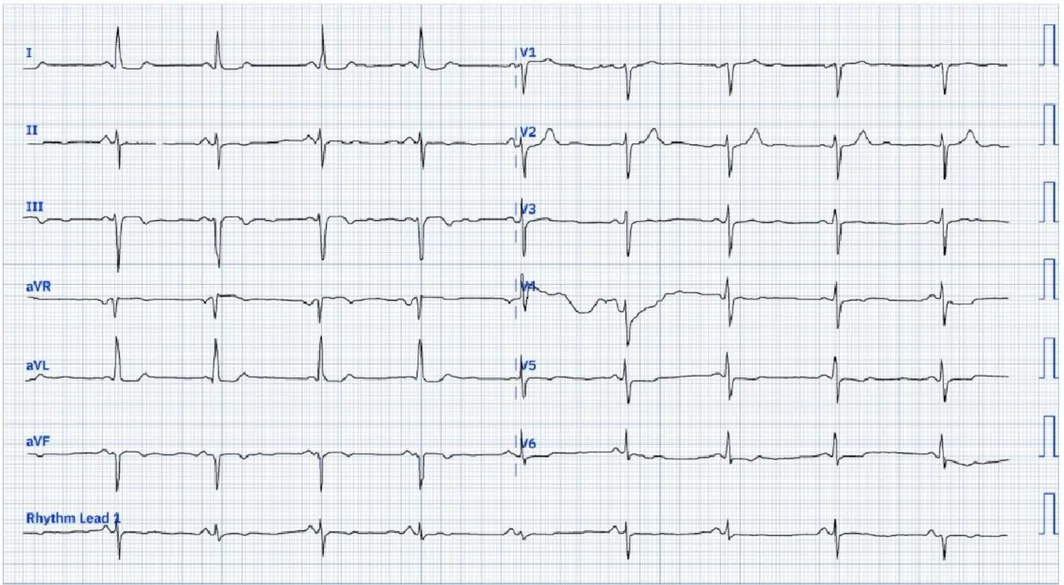
Initial electrocardiogram (ECG) findings suggestive of myocardial ischemia.

Electrocardiography showed sinus rhythm with T‐wave inversion in the inferior leads (II, III, aVF) and slight ST segment depression in the lateral leads (I, aVL, V5–V6). This indicated myocardial ischemia (Figure 1).

The initial high‐sensitivity troponin T was 15.8 ng/L, marginally above the commonly cited 99th‐percentile upper reference limit of approximately 14 ng/L for this assay. On repeat testing at 24 h it had risen markedly to 747.0 ng/L, a delta of 731.2 ng/L, confirming acute myocardial injury; the full laboratory results are given in Table 1. On the same admission electrocardiogram, the PMcardio application flagged ischemic changes, an output that was concordant with the treating team's interpretation. AI‐based ECG analysis has been reported to improve recognition of ischemic changes that can be overlooked on conventional interpretation, although diagnostic performance was not formally assessed in this single case. The electrocardiogram showed sinus rhythm at 66 beats per minute with T‐wave inversion in the inferior leads and mild ST‐segment depression in the lateral leads.

**TABLE 1:** Laboratory findings on admission (Day 1) and 24 h later (Day 2).

Entity	Unit	Day 1	Day 2
Sodium	mmol/L	138.1	135.3
Potassium	mmol/L	4.03	4.04
Chlorine	mmol/L	105.4	100.8
Bicarbonate	mmol/L	23.3	21.3
Calcium	mmol/L	2.30	2.37
Phosphorus	mmol/L	1.03	1.18
Magnesium	mmol/L	0.74	0.75
Creatine kinase (CK)	U/L	85	417
Troponin T	ng/L	15.8	747.0

### 3.2 Blood Results

Table 1 depicts the laboratory findings obtained at the time of presentation and following 24 h. Tests revealed a marked rise in levels of troponin T and creatine kinase, pointing towards acute myocardial infarction, while no changes were noted in other biochemical markers.

### 3.3 Timeline

In accordance with the CARE reporting guidance, the clinical course is summarized chronologically [5]. Approximately 2 weeks before admission, the patient developed progressive exertional chest pain radiating to the left arm and jaw. On the day of admission (Day 1), he presented to the emergency department with a blood pressure of 176/105 mmHg and a heart rate of 66 beats per minute; the 12‐lead ECG showed sinus rhythm with inferior T‐wave inversion and mild lateral ST‐segment depression without ST‐elevation, the PMcardio application flagged ischemic changes on this ECG, and the initial high‐sensitivity troponin T was 15.8 ng/L. On the same day the presentation was categorized as high‐risk non‐ST‐elevation acute coronary syndrome and an early invasive strategy was undertaken: after intravenous heparin and acetylsalicylic acid loading and premedication for a known contrast allergy, urgent coronary angiography demonstrated a thrombotic occlusion of the proximal right coronary artery, which was treated by percutaneous coronary intervention with two drug‐eluting stents. By 24 h (Day 2), high‐sensitivity troponin T had risen to 747.0 ng/L, and nonsustained ventricular tachycardia occurred twice and was managed with bisoprolol. During the remainder of the admission, transthoracic echocardiography showed preserved biventricular function, dual antiplatelet and secondary‐prevention therapy were established, early cardiac rehabilitation was commenced, and the patient was discharged on guideline‐directed therapy.

### 3.4 Therapeutic Intervention

Given that the patient exhibited symptoms of ischemia, dynamic ECG changes and a rising high‐sensitivity troponin, therapeutic management followed current guidelines for acute coronary syndrome [1, 6]. Because these features identified a high‐risk non‐ST‐elevation acute coronary syndrome, an early invasive strategy was pursued. He received intravenous heparin (5000 IU) and an intravenous loading dose of acetylsalicylic acid (500 mg, administered as lysine acetylsalicylate in keeping with European emergency practice), followed by urgent coronary angiography.

Angiography demonstrated a thrombotic occlusion of the proximal right coronary artery, which was treated by percutaneous coronary intervention with implantation of two drug‐eluting stents and restoration of coronary flow. Dual antiplatelet therapy with aspirin and ticagrelor was initiated, together with a beta‐blocker, high‐intensity statin, and an ACE inhibitor, in line with guideline‐directed therapy [6, 7]. Because of the patient's history of contrast allergy, he received prophylactic methylprednisolone (Solu‐Medrol) before the procedure.

## 4 Conclusion and Results (Outcome and Follow‐Up)

This patient was found to have had a favorable clinical course after undergoing PCI with successful reperfusion of the proximal right coronary artery and placement of two drug‐eluting stents. There were findings of sinus rhythm and inverted T waves in the inferior leads together with mild ST segment depression as detected from the PMCardio app and attending cardiologists. There was also normal left and right ventricular systolic function (preserved left ventricular ejection fraction) together with trace mitral and tricuspid regurgitation and absence of pulmonary hypertension on transthoracic echocardiography. The patient tolerated the procedure well especially when given premedication to counter his contrast allergy. He was discharged on aspirin 80 mg once daily, ticagrelor 90 mg twice daily, bisoprolol 2.5 mg once daily, ezetimibe/rosuvastatin 10/40 mg once daily, ramipril 2.5 mg once daily, omeprazole 40 mg once daily, levothyroxine 125 mcg once daily, a nicotine‐replacement patch 21 mg/24 h, and paracetamol 1 g as needed for pain (maximum 4 g per day).

The adherence and tolerance to intervention were evaluated at the stage of hospitalization using direct observations and consultation with patients. The patient has received thorough information concerning the timing of intake, antiplatelet drug regimen, as well as changes in lifestyle without any acute adverse reactions. Early cardiac rehabilitation program that includes physical activity, nutrition, and psychological support has been started by following the guidelines [8]. The patient was aware of the necessity to follow all recommendations and was motivated to do that. Nonsustained ventricular tachycardia after reperfusion was diagnosed twice but the patient was successfully treated with low doses of bisoprolol.

Beyond guideline‐directed secondary prevention, newer cardiometabolic agents such as sodium‐glucose cotransporter‐2 inhibitors and glucagon‐like peptide‐1 receptor agonists are of growing interest after acute coronary syndrome, although they were not indicated in this nondiabetic patient and their role in the acute post‐ACS setting remains under investigation [9, 10]. Follow‐up in this report was limited to the index hospitalization, and longer‐term outcome data were not available.

## 5 Discussion

### 5.1 Clinical Context and Diagnostic Challenge

The current case demonstrates difficulties in the diagnosis of ACS in patients whose presentation does not meet STEMI criteria and whose preliminary results are inconclusive. For our patient, the presence of mild ECG changes together with an initially borderline troponin caused diagnostic uncertainty during the first phase of evaluation. The PMcardio output was concordant with the treating team's recognition of ischemic changes at a time when the diagnosis was not yet established, and urgent coronary angiography with revascularization followed. One of the key issues of emergency cardiology is the detection of the patient with acute coronary artery occlusion who does not satisfy criteria of STEMI. Quite a significant number of patients with NSTE‐ACS have occlusive lesions, and their treatment is deferred and less effective [4]. The routine methods of diagnosis that include ECG reading, serial biomarkers, and risk scores can fail to detect such patients.

### 5.2 Performance of PMcardio in NSTEMI Ischemia Detection

PMcardio incorporates a dedicated occlusion myocardial infarction (OMI) model, known as the Queen of Hearts, that interprets a photographed 12‐lead ECG using deep learning [4]. In its international development and validation study, this OMI model was derived from 18,616 ECGs from 10,543 patients with suspected ACS and achieved an area under the curve of 0.938, an accuracy of 90.9%, a sensitivity of 80.6%, and a specificity of 93.7%, outperforming conventional STEMI criteria and matching expert ECG readers [4]. This OMI model is distinct from the general PMcardio interpretation system evaluated in primary care (AMSTELHEART‐1), which showed a sensitivity of 86% and a specificity of 92% for major ECG abnormalities and near‐perfect performance for atrial fibrillation, but limited sensitivity for markers of past ischemia and reduced reliability with poor‐quality ECGs [3]. In a retrospective study of high‐risk non‐ST‐elevation ACS, Carvalho et al. reported that the OMI model improved rule‐in specificity and positive predictive value (both 78%) and reduced false‐positive catheterization‐laboratory activations compared with standard care, whereas rule‐out sensitivity on the initial ECG was limited (58%) and improved with serial ECGs [11]. In a separate single‐center retrospective cohort (AERO‐ACS), Choi et al. found a sensitivity of 86.5% and a specificity of 82.2% for angiographic OMI, compared with a sensitivity of 54.1% for STEMI criteria, and 100% sensitivity for STEMI‐OMI [12]. Both studies were retrospective and subject to selection bias, and neither established a causal effect of the tool on patient outcomes.

Taken together, these studies suggest that the PMcardio OMI model may support OMI detection and triage in NSTEMI or nondiagnostic ECGs, but serial ECGs, biomarkers, and clinical judgment remain necessary [11, 12].

### 5.3 Case Report: AI‐Assisted Ischemic Changes Detection

In this particular case, the PMcardio algorithm detected subtle ischemic changes in the inferolateral leads in the ECG, which did not qualify for STEMI, but which were accompanied with a slightly elevated troponin and necessitated immediate angiography and reperfusion. It is in agreement with the data on validation in primary care demonstrating high sensitivity and specificity of the PMcardio algorithm in detecting major ECG abnormalities (including ischemia), as well as the almost perfect results in detecting atrial fibrillation [3]. Moreover, the described case reflects the issue of the majority of ACS cases lacking classical STEMI changes in ECG and a significant number of NSTEMI patients having OMIs that are associated with poor prognosis [4]. The recently developed AI‐based OMI ECG model developed using an international population sample of ACS patients performed better than the STEMI criteria in terms of sensitivity and accuracy and was comparable to that of ECG specialists, thus facilitating identification of OMI cases in the early stages, thus making the process of triage for urgent revascularization more effective [4]. These findings are consistent with contemporary chest‐pain guidelines, which recommend prompt ECG acquisition, high‐sensitivity troponin testing and formal risk assessment to identify high‐risk patients who may require an invasive strategy [2, 6]. In addition to ACS there is also research of deep learning models, which are able to interpret 12‐lead ECG. This shows how artificial intelligence can diagnose different abnormalities in ECG [13]. This falls under the concept of “high performance medicine,” where artificial intelligence assists medical professionals by increasing the effectiveness and speed of cardiology practice with fairness [14]. For example, in the current case the use of an ECG artificial intelligence tool via smartphone represents a kind of assistant. An increase in the sensitivity to ischemia diagnosis combined with the efficient work of existing guidelines. It is important to remember, however, that the final diagnosis, treatment, and other treatment methods (e.g., anticoagulation therapy, cardiac rehabilitation), remain the domain of medical professionals.

### 5.4 Challenges and Ethical Considerations

Even though AI instruments, such as PMcardio, are very promising, several limitations have to be taken into account. First, the efficiency of algorithms used in AI may depend on the quality of ECG recording, as well as patients' demographic information and comorbidity factors. Moreover, the generalizability of the results obtained using PMcardio in different clinical cases is unclear, and there is a need for conducting further validation studies among wider groups of patients and in other healthcare settings [13]. Second, ethical issues that may arise while implementing the AI instrument in practice, such as the possible over‐reliance on algorithms and consequences of incorrect diagnosis, should be considered [14]. The use of AI in medicine should supplement clinical experience, not substitute it. In the case described above, PMcardio assisted in making decisions, but cardiac catheterization was still needed to confirm the diagnosis.

Several further limitations should be emphasized. This is a single case in which the PMcardio output was concordant with the final angiographic diagnosis; concordance in one patient cannot demonstrate improved sensitivity, earlier reperfusion or better outcomes, and provides no information on false positives or false negatives. AI‐based ECG tools are susceptible to spectrum and selection bias, to miscalibration in populations that differ from the development cohorts, and to automation bias, whereby clinicians may defer inappropriately to the algorithm. Performance also depends on the quality of the photographed ECG image. The output described here reflects the application as used at the point of care, and the original on‐screen report, the exact confidence score and the software version were not available for inclusion. Robust conclusions will require prospective, comparative studies of human‐AI workflows reported according to established standards for the early clinical evaluation of AI decision‐support systems [15].

## 6 Conclusions

This case illustrates a non‐ST‐elevation acute coronary syndrome in which an artificial‐intelligence ECG output was concordant with the treating team's interpretation and the final angiographic diagnosis. It highlights the potential supportive role of tools such as PMcardio in emergency cardiology, while underscoring that the diagnosis was confirmed by angiography rather than by the algorithm. Prospective studies are needed to determine whether such tools improve diagnostic timeliness and patient outcomes, and how they perform across clinical settings.

## Author Contributions

**Muhammad Sharjeel Abbas:** resources, supervision. **Bipin Chaurasia:** validation, visualization, supervision, writing – review and editing. **Priyavardhan Mishra:** software. **Rinaldo Lauwers:** validation. **Atif Qureshi:** visualization. **Ramzi Zeidan:** formal analysis. **Murtaja Satea:** visualization. **Kim Wouters:** conceptualization, investigation, writing – original draft, methodology.

## Funding

The authors have nothing to report.

## Ethics Statement

We confirm that we have read the Journal's position on issues involved in ethical publication and affirm that this report is consistent with those guidelines.

## Consent

Written consent was provided by the patient, included in this case report, for the publication of the details of their medical case and associated images. The patient was assured of confidentiality and that no identifying information would be disclosed.

## Conflicts of Interest

The authors declare no conflicts of interest.

## Data Availability

The data that support the findings of this study are available from the corresponding author upon reasonable request.

## References

[1] R. A. Byrne , X. Rossello , J. J. Coughlan , et al., “2023 ESC Guidelines for the Management of Acute Coronary Syndromes,” European Heart Journal 44, no. 38 (2023): 3720–3826. 37622654 10.1093/eurheartj/ehad191

[2] M. Gulati , P. D. Levy , D. Mukherjee , et al., “AHA/ACC/ASE/CHEST/SAEM/SCCT/SCMR Guideline for the Evaluation and Diagnosis of CHEST Pain: A Report of the American College of Cardiology/American Heart Association Joint Committee on Clinical Practice Guidelines,” Circulation 144, no. 22 (2021): e368–e454. 10.1161/CIR.0000000000001029 34709879

[3] J. C. L. Himmelreich and R. E. Harskamp , “Diagnostic Accuracy of the PMcardio Smartphone Application for Artificial Intelligence‐Based Interpretation of Electrocardiograms in Primary Care (AMSTELHEART‐1),” Cardiovascular Digital Health Journal 4, no. 3 (2023): 80–90. 37351331 10.1016/j.cvdhj.2023.03.002 PMC10282008

[4] R. Herman , H. P. Meyers , S. W. Smith , et al., “International Evaluation of an Artificial Intelligence‐Powered Electrocardiogram Model Detecting Acute Coronary Occlusion Myocardial Infarction,” European Heart Journal ‐ Digital Health 5, no. 2 (2024): 123–133. 38505483 10.1093/ehjdh/ztad074 PMC10944682

[5] J. J. Gagnier , G. Kienle , D. G. Altman , et al., “The CARE Guidelines: Consensus‐Based Clinical Case Reporting Guideline Development,” BMJ Case Reports 2013 (2013): bcr2013201554. 10.1136/bcr-2013-201554 PMC3822203 24155002

[6] S. V. Rao , M. L. O'Donoghue , M. Ruel , et al., “2025 ACC/AHA/ACEP/NAEMSP/SCAI Guideline for the Management of Patients With Acute Coronary Syndromes: A Report of the American College of Cardiology/American Heart Association Joint Committee on Clinical Practice Guidelines,” Circulation 151, no. 13 (2025): e771–e862. 40014670 10.1161/CIR.0000000000001309

[7] P. E. P. Carvalho , D. M. Gewehr , B. R. Nascimento , et al., “Short‐Term Dual Antiplatelet Therapy After Drug‐Eluting Stenting in Patients With Acute Coronary Syndromes: A Systematic Review and Network Meta‐Analysis,” JAMA Cardiology 9, no. 12 (2024): 1094–1105. 39382876 10.1001/jamacardio.2024.3216 PMC11581547

[8] A. A. Damluji , C. R. Tomczak , S. Hiser , et al., “Benefits of Cardiac Rehabilitation: Mechanisms to Restore Function and Clinical Impact,” Circulation Research 137, no. 2 (2025): 255–272. 40608851 10.1161/CIRCRESAHA.125.325705 PMC12233141

[9] P. Karakasis , D. Patoulias , G. Kassimis , et al., “Therapeutic Potential of Sodium‐Glucose Co‐Transporter‐2 Inhibitors and Glucagon‐Like Peptide‐1 Receptor Agonists for Patients With Acute Coronary Syndrome: A Review of Clinical Evidence,” Current Pharmaceutical Design 30, no. 27 (2024): 2109–2119. 38910481 10.2174/0113816128304097240529053538 PMC11475097

[10] P. Karakasis , N. Fragakis , K. Kouskouras , T. Karamitsos , D. Patoulias , and M. Rizzo , “Sodium‐Glucose Cotransporter‐2 Inhibitors in Patients With Acute Coronary Syndrome: A Modern Cinderella?,” Clinical Therapeutics 46, no. 11 (2024): 841–850. 38991865 10.1016/j.clinthera.2024.06.010

[11] P. E. P. Carvalho , W. Belzer , D. L. Pollmann , et al., “AI‐Enhanced Electrocardiogram for Detection of Occlusive Myocardial Infarction in High‐Risk Non‐ST‐Segment Elevation Acute Coronary Syndrome,” JACC Advances 5, no. 4 (2026): 102663. 41887018 10.1016/j.jacadv.2026.102663 PMC13053991

[12] J. W. H. Choi , V. Torelli , A. Silverman , et al., “AI‐Enhanced Recognition of Occlusions in Acute Coronary Syndrome (AERO‐ACS): A Retrospective Study,” Coronary Artery Disease 37, no. 1 (2026): 39–45. 40705383 10.1097/MCA.0000000000001555

[13] A. H. Ribeiro , M. H. Ribeiro , G. M. M. Paixão , et al., “Automatic Diagnosis of the 12‐Lead ECG Using a Deep Neural Network,” Nature Communications 11, no. 1 (2020): 1760. 10.1038/s41467-020-15432-4 PMC7145824 32273514

[14] E. J. Topol , “High‐Performance Medicine: The Convergence of Human and Artificial Intelligence,” Nature Medicine 25, no. 1 (2019): 44–56. 10.1038/s41591-018-0300-7 30617339

[15] B. Vasey , M. Nagendran , B. Campbell , et al., “Reporting Guideline for the Early Stage Clinical Evaluation of Decision Support Systems Driven by Artificial Intelligence: DECIDE‐AI,” BMJ 377 (2022): e070904. 35584845 10.1136/bmj-2022-070904 PMC9116198

